# Inbreeding strategies under social and sexual complexity: insights from female and male guppies

**DOI:** 10.1093/beheco/arag017

**Published:** 2026-03-04

**Authors:** Amanda Viving, Léa Daupagne, David Wheatcroft, Niclas Kolm, John L Fitzpatrick

**Affiliations:** Department of Zoology, Stockholm University, Svante Arrhenius väg 18B, Stockholm 10691, Sweden; Department of Zoology, Stockholm University, Svante Arrhenius väg 18B, Stockholm 10691, Sweden; Department of Zoology, Stockholm University, Svante Arrhenius väg 18B, Stockholm 10691, Sweden; Department of Zoology, Stockholm University, Svante Arrhenius väg 18B, Stockholm 10691, Sweden; Department of Zoology, Stockholm University, Svante Arrhenius väg 18B, Stockholm 10691, Sweden

**Keywords:** inbreeding avoidance, precopulatory mate choice, social complexity, guppy

## Abstract

Animals are typically expected to avoid mating with relatives due to the costs associated with incestuous matings. Yet for more than 4 decades, theoretical models have consistently suggested that animals may tolerate, or even prefer, mating with relatives under a broad range of conditions. However, empirical studies that evaluate inbreeding strategy under alternative social and sexual contexts remain scarce. Here, we investigate how experimental variation in sexual and social complexity influence precopulatory inbreeding avoidance behaviors in the guppy *Poecilia reticulata*, a species known to experience inbreeding depression. In an integrated set of experiments, we examined if sexual and affiliative behaviors of virgin and experienced females and males were differentially directed towards either related or unrelated individuals. In simple social context, neither virgin nor experienced females or males showed a preference for related or unrelated partners. In more sexually and socially complex free-swimming arenas, females appeared to respond slightly more to related males, but this effect was not statistically significant once corrected for multiple comparisons; male preference remained unchanged. Overall, these findings challenge previous reports of preference shifts between virgin and experienced female guppies and suggest that inbreeding avoidance behaviors may be less prevalent in complex social environments than previously thought.

## Teaser

Although animals are often expected to avoid inbreeding, theory suggests mating with relatives can sometimes be beneficial. We tested how social and sexual complexity affects guppies' mating decisions. In simple setups, neither females nor males preferred relatives or nonrelatives. In more naturalistic, free-swimming arenas, females showed a weak tendency to respond more to related males, while males showed no preference. These results suggest that inbreeding avoidance may be limited in guppies.

## Introduction

The question of why individuals mate with relatives when inbreeding has clear fitness costs (the so-called *inbreeding paradox*) has recently gained considerable theoretical and empirical interest (Reid et al. 2015; Townsend et al. 2019; de Boer et al. 2021; Montecinos et al. 2021; Dorsey and Rosenthal 2023). Potential solutions to the inbreeding paradox have historically focused on the relative costs and benefits of inbreeding, particularly in terms of inclusive fitness. Specifically, inbreeding might be favored through kin selection, provided the potential negative effects of inbreeding are offset by the benefits associated with increasing the reproductive success of related individuals. Thus, choosing to mate with kin has been proposed as an adaptative strategy that enhances an individual's indirect inclusive fitness (Parker 1979; Waser et al. 1986; Lehmann and Perrin 2003; Kokko and Ots 2006; Szulkin et al. 2013; Duthie and Reid 2016). Recently, Dorsey and Rosenthal (2023) offered a novel solution to the inbreeding paradox that focuses on the potential benefits of kin affiliation in sexual versus nonsexual situations. While sexual affiliation with relatives is often associated with fitness costs (ie, through inbreeding depression), animals often benefit by interacting with relatives in nonsexual contexts. For instance, in species living in groups, kin preference can translate into fitness benefits by increasing individual growth (eg, Brown and Brown 1996; Thünken et al. 2016), potentially mitigating inbreeding costs. Because the benefits of nonsexual interactions with relatives require affiliative behaviors among kin, the evolution of mechanisms that reduce sexual interactions with kin may be constrained (Dorsey and Rosenthal 2023), particularly when affiliative behaviors are selected at the adult phase. Yet, research on how sexual and nonsexual behaviors vary among social partners based on relatedness remains limited, as most studies focus on kin preferences during either sexual (eg, mate choice) or nonsexual (eg, juvenile and/or parental) life stages.

Inbreeding strategies can also be shaped by a range of factors, further complicating our ability to understand how preference for kin affiliation during nonsexual situations may constrain inbreeding avoidance during mate choice. For example, experimental approaches typically examine sexual behaviors without considering nonsexual behaviors and rarely examine inbreeding avoidance in complex social environments. Most studies employ a simple, dichotomous choice design, which may not fully capture the social dynamics found in natural systems (Carleial et al. 2020). Strong precopulatory mate preference may be limited or be overridden through intra-sexual competition (Forsgren 1997; Kangas and Lindström 2001; Sih et al. 2002; Candolin 2004) or sneak matings (Brockmann 2001; Shuster and Wade 2003). Indeed, previous studies show that an individual's sexual and social behavior during free interactions in complex social settings may shift depending on the sex ratio, the number of surrounding conspecifics, and whether those conspecifics are related or not (Carazo et al. 2014; Martin and Long 2015; Lymbery and Simmons 2017; Daniel and Williamson 2020). Additionally, studies exploring the effects of inbreeding avoidance within simple versus complex social environments usually do not account for key biological factors that may influence an individual's propensity for inbreeding avoidance (de Boer et al. 2021). First, responses to inbreeding avoidance are predicted to be sex-specific, with females expected to exert stronger mate choice, while males are generally assumed to be less choosy (Blouin and Blouin 1988; Gerlach and Lysiak 2006; de Boer et al. 2021). Second, mating status may influence inbreeding avoidance, as virgin individuals are expected to mate indiscriminately to ensure reproductive assurance, while mated individuals are expected to be more selective, with choosiness increasing with mating experience (Jennions and Petrie 1997, 2000; Kokko and Ots 2006; Tanner et al. 2019). Indeed, evidence suggests that females tend to “trade-up” in genetic quality during sequential mate choice by mating with more compatible (eg Bradley et al. 1990; Svensson et al. 2010) or attractive males in later mating opportunities (eg Pitcher et al. 2003). In the context of inbreeding, this theory is supported by a recent study from Daniel and Rodd (2016) showing that virgin female guppies show no kin discrimination during their first mating episode, but avoid incest after they were exposed to, and mated with, a single male for a 24 h period. This evidence is, however, challenged by 2 meta-analyses (de Boer et al. 2021; Richardson and Zuk 2023), which indicate that mating status generally has limited influence on inbreeding avoidance and female choosiness, while also highlighting the lack of studies explicitly considering mating status and emphasizing the need for its inclusion in future research (de Boer et al. 2021; Richardson and Zuk 2023).

Here, we investigated how sex, mating status, and the complexity of the sexual-social environment influence behavioral affiliations for related versus unrelated individuals in both sexual and nonsexual contexts in the guppy (*Poecilia reticulata*). The guppy is an internally fertilizing, live-bearing freshwater fish, with a nonresource based, polygynandrous mating system. In the wild, guppies experience dry seasons that may create isolated small populations, where relatives are more likely to encounter one another, increasing the risk of inbreeding (Griffiths and Magurran 1997b). Inbreeding depression can be severe in guppies, as inbred offspring exhibit reduced survival rates (Nakadate et al. 2003; Van Oosterhout et al. 2007 ), lower courtship displays and sexual coloration (Van Oosterhout et al. 2003), and diminished sperm counts (Zajitschek and Brooks 2010). Although females are generally the choosier sex, males may also display mating preferences, as they often encounter several females simultaneously (Houde 1997c). Moreover, sperm production requires a refractory period between successive matings (Pilastro and Bisazza 1999), prompting males to allocate sperm to the highest-quality female when reserves are limited (Andersson 1994; Dosen and Montgomerie 2004). Guppies express mating preference towards traits such as body size, female receptivity and male body coloration (Houde 1997b; Auld et al. 2016; Corral-López et al. 2017), and can also base their mating decision through kin recognition mechanisms (Griffiths and Magurran 1999; Penn and Frommen 2010). Kin recognition can be achieved through phenotype matching, where an individual uses a template of its own phenotype (ie self-referencing) and/or that of its conspecifics with whom it develops, such as its broodmates (ie familiarity) to later evaluate others (Penn and Frommen 2010). In the guppy, Griffiths and Magurran (1997a) first demonstrated kin recognition via familiarity: both juvenile and adult females preferentially shoaled with familiar over unfamiliar conspecifics, but showed no preference for unfamiliar kin. More recently, Daniel and Rodd (2021) provided evidence that guppies also use self-referencing to recognize kin by comparing olfactory phenotypes of conspecifics to their own. Given the risk of inbreeding in the wild, the associated fitness costs, and guppies' ability to distinguish kin through various mechanisms, it is expected that guppies have evolved precopulatory inbreeding avoidance through mate choice (Daniel and Rodd 2016). However, previous studies have shown mixed results and lacked ecologically relevant conditions (Viken et al. 2006; Pitcher et al. 2008; Guevara-Fiore et al. 2010; Zajitschek and Brooks 2010; Daniel and Rodd 2016). In particular, while Daniel and Rodd (2016) demonstrated that mated female guppies are more likely to avoid inbreeding than virgin females, this effect was only evident after fish were reared in constant visual contact with kin for 100 to 120 d, a situation that is unlikely to occur in nature. In addition, guppies form stable social networks (Croft et al. 2004; Croft et al. 2006), which can promote preferential interactions among kin in nonsexual contexts. Such kin-based social bonds may provide cooperative benefits, such as increased survival or access to resources (eg Liévin-Bazin et al. 2019), potentially influencing the tradeoff between affiliating with kin and avoiding inbreeding. If these nonsexual interactions are particularly beneficial among adult relatives, selection may act against strict precopulatory inbreeding avoidance in favor of maintaining social cohesion. By using individuals of both sexes with different mating statuses within environments of varying sexual-social complexity, this study aimed to identify factors that may constrain or modify the costs and benefits associated with inbreeding. We predicted that (i) females would exhibit stronger inbreeding avoidance than males, (ii) virgins would be less choosy than experienced individuals, especially among females, (iii) individuals would preferentially interact with unrelated individuals in a sexual context, whereas, such preference would not be observed or reversed in a nonsexual context.

## Methods

### Experimental fish and rearing conditions

Fish used in this study were descendents from an initial population of >500 guppies caught from a high-predation area of the Quare River, Trinidad, in 1998. Stock populations were maintained in groups of several hundred outbred fish, with regular movement of fish among stock populations to minimize inbreeding risk. At the time of this study, fish were held in the lab for ∼50 to 75 generations (assuming 2 to 3 generation per year). Although the stock population was founded from several hundred wild individuals and maintained at large sizes for many generations, some degree of background inbreeding due to genetic drift cannot be ruled out, which may slightly reduce the magnitude of relatedness contrasts. From these stock populations, we generated 24 distinct families of full sibling offspring, each generated through an independent cross between a single virgin female and male guppy. To further reduce the risk of inbreeding, 24 females and 24 males were randomly selected from 2 different stock tanks, with each pair formed by combining a female from 1 tank and male from the other. These pairs were then housed in 4-L tanks until they produced their first brood. The fry from each brood were housed in groups of no more than 6, and were separated into sex-specific tanks when males reached sexual maturity (at ∼2 to 3 mo). This method resulted in full siblings (*r* ∼ 0.5) and unrelated individuals (*r* ∼ 0). The tanks were visually isolated from the opposite sex, as male color patterns can influence future mate choice decisions in females (Breden et al. 1995; Hughes et al. 1999). The fish bred for this experiment were 9 to 11 mo old at the time of the experiment. This prolonged separation between opposite-sex relatives is unlikely to impair kin recognition as guppies also exhibit phenotype matching through self-referencing (ie, exposure to phenotypic cues of kinship is not a prerequisite for kin recognition in guppies, Daniel and Rodd 2021). The fish were kept on a 12:12 light:dark cycle at 26 to 28 °C and fed dry flakes and Artemia.

### Experimental procedure

**Figure 1:**
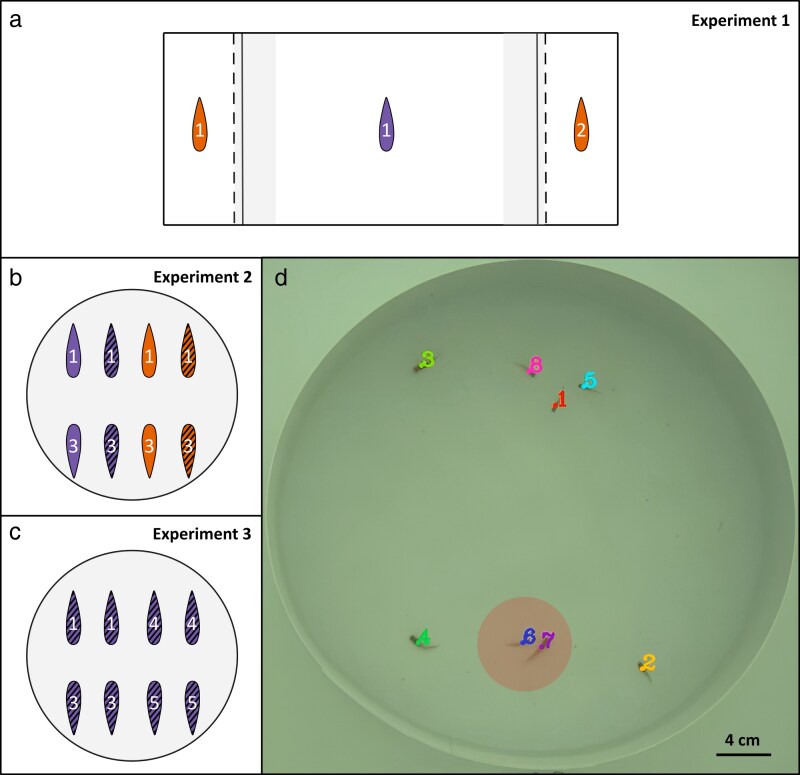
Experimental settings used in the study. In panels a, b, and c, females are shown in purple, males in orange, and the numbers correspond to families. Striped patterns indicate sexually experienced individuals, while plain (unstriped) patterns represent virgin individuals. a) Schematic representation of a dichotomous mate choice tank (Experiment 1). The focal individual in the main compartment (female or male, virgin or experienced) faced 2 stimulus individuals (related and unrelated) of the opposite-sex placed at random in the left and right stimulus compartments. Two opaque barriers (solid black lines) prevented visual contact between the stimulus compartments and the chooser individual in the main compartment. After habituation, the opaque dividers were lifted and mate choice was assessed by recording the time the focal individual spent in either the left or right association zone (24 cm × 5 cm, here shown in gray areas). b) Schematic representation of a free-swimming mate choice arena (Experiment 2). Four individuals from a single family, representing both sexes and mating statuses, were introduced to interact with 4 individuals from a unrelated family of identical composition. Prior to this interaction, the 2 family groups had no previous exposure to one another. c) Schematic representation of a free-swimming social interactions arena (Experiment 3). Two same-sex individuals from a single family were introduced to interact with 2 individuals from each of 3 unrelated families. d) Example of a tracked video generated by idtracker.ai, ie an original input video overlayed with the individual trajectories and labels, enabling identification of individuals engaging in sexual behaviors. *idtracker.ai* provides the position of the centroid of each individual in pixel coordinates for every frame. Individuals were considered associated when located within a 3.52 cm radius of each other (see Methods “Experiment 2: free-swimming mate choice” section); eg individual 7 is within individual 6's association zone (redarea), indicating an association between the pair at that frame. Each focal individual tested in Experiment 1 was also tested in Experiment 2 and 3.

The role of relatedness in shaping sexual and social interactions among guppies was assessed in 3 separate and successive experiments (Fig. 1). Each focal individual was tested in each experimental setting. In Experiment 1 (dichotomous mate choice), a focal individual was presented with 2 size-matched stimuli individuals of the opposite sex that differed in their relatedness (related versus unrelated, Fig. 1a). In Experiment 2, 4 individuals from 1 family (2 females, 2 males) were placed together with 4 individuals from another family (2 females, 2 males) in a free-swimming arena and reproductive behaviors were recorded (Fig. 1b). In Experiment 3, 2 same-sex individuals from 1 family were placed together with 2 same-sex individuals from 3 other families (ie, forming groups of 8 fish of the same sex, Fig. 1c). The experiments were divided equally into morning and afternoon sessions.

#### Acclimation and mating status definition

All fish were virgins at the start of the experiment. Each fish was housed in a 7-L acclimation tank with a same-sex sibling for 4 d prior to Experiment 1. This setup was designed to familiarize the fish with a white gravel background similar to that used in the experimental trials. One day prior to Experiment 1, a focal fish (either male or female) was housed together in a 4-L tank with 1 individual from a different family of the same sex (same-sex housing) to ensure they remained virgin, or, of opposite-sex (opposite-sex housing) to provide individuals with mating experience. These pairings were between unrelated families with no prior experience, and each pair was kept together for approximately 24 h to allow sufficient time for mating. Based on the protocol established by Daniel and Rodd (2016), mating typically occurs within this time period. After the housing period, each fish was transferred to an individual 4-L tank enriched with plants and white gravel, where they remained for the duration of the experimental period.

#### Experiment 1: dichotomous mate choice experiment

To test if mating status and sex influenced mate preferences, we first presented fish with a dichotomous choice of a related and unrelated potential partner. The dichotomous mate choice was conducted in 40 × 24 × 20 cm rectangular tanks divided into 3 compartments: 1 main compartment (27 × 24 × 20 cm) which housed the focal individual (female or male, virgin or experienced), and 2 smaller compartments (each 6 × 24 × 20 cm) which housed the 2 opposite-sex stimulus individuals, one of which was related to the focal individual and the other unrelated (see Fig. 1a). The stimulus individuals were size-matched by eye and the placement of the related and unrelated individuals in the smaller compartments was randomized. All tanks were filled to a depth of 5 cm. A transparent plastic barrier with small openings (allowing water flow between chambers) separated stimulus individuals from the focal individual, allowing the focal individual to use both olfactory and visual cues to assess stimulus individuals during the trials (Houde 1997a, 1997c; Shohet and Watt 2004; Guevara-Fiore et al. 2009). Before each trial, the individuals were given 30 min to acclimatize to their environment. During this time, visual contact between the main and stimulus chambers was prevented by 2 opaque dividers. After habituation, the opaque barriers were removed and mate choice was assessed by recording the time the focal individual spent in each association zone, an area of 5 cm in front of the respective stimulus compartments (24 × 5 cm, see Fig. 1a). The time individuals spend in an association zone is a commonly used measure of mating preference that is predictive of mate preference during sexual interactions (Dougherty 2020). Trials were recorded using a camera (Point Gray Grasshopper 3 4.1 megapixel camera with Fujinon CF25HA-1 lens) directly placed 1.5 m above the experimental tanks. To remove olfactory cues between trials, each tank was drained and refilled with fresh water following each trial.

In total, 96 trials were performed, testing 96 individuals (48 males, 48 females) with different mating status (24 virgin males, 24 experienced males, 24 virgin females and 24 experienced females). In a few cases, the focal fish went under the opaque barrier during the acclimation period (*n* = 7, 3 virgin males, 2 experienced males and 2 experienced females) so the recordings were excluded from the analysis. This resulted in a final sample size of 89 trials being included in the statistical analysis. From the recording, mate preference was assessed using an automated tracking software (EthoVision XT 17, Noldus Information Technology). Video analyses were performed by a single observer (A.V.) blind to both mating status and relatedness.

#### Experiment 2: free-swimming mate choice

The free-swimming mate choice was assessed in cylinder arenas (36 cm in diameter), containing 8 individuals, each of whom had been tested as a focal subject in the dichotomous mate choice experiment the previous day (see Fig. 1b). In each trial, 4 individuals from a single family, representing both sex and mating statuses, were allowed to freely interact with 4 individuals from an unrelated family of identical composition. Each family group included 2 individuals that had been housed together during the acclimation period (Acclimation and mating status definition), meaning that same-sex siblings re-encountered one another during these trials. The 2 families had not experienced one another prior to the trial. The individuals were acclimated in opaque isolated chambers (5 × 6 cm) within the cylinder arena for 15 min, which were then lifted, and the behaviors of the individuals were recorded for 30 min.

Behavioral scoring was assessed using Behavioral Observation Research Interactive Software *BORIS* (Friard and Gamba 2016). During observations, tracked videos generated by *idtracker.ai* (Romero-Ferrero et al. 2019) were simultaneously playing, allowing the identification of individuals engaging in sexual behaviors. In females, we scored 3 sexual behaviors often expressed sequentially: orienting behavior (turning and facing the displaying male), approaching behavior (moving towards the displaying male) and gliding behavior (moving in a smooth gliding fashion towards the male) (Houde 1997c). Of these, gliding behavior is the clearest indicator of female preference as it is commonly observed immediately prior to mating (Liley 1966; Houde 1997c). In males, we scored the time spent pursuing the female (following the female, <2 body lengths away), the number of sigmoid displays (quivering with an S-shaped arch of the body to the side or in front of the female), the number of successful matings, and the number of forced mating attempts (an alternative mating strategy where the male moves behind the female and thrusts his gonopodium at the female's gonopore) (Houde 1997c). In total, 12 trials were performed (each consisting of 8 individuals). Video analyses were conducted by a single observer (A.V.) blind to both mating status and relatedness.

We also examined sexual and social interactions within the arenas by recording the spatial proximity between each pair of individuals. Using identity-registered centroid trajectories produced by *idtracker.ai* version 5.2 11. (Romero-Ferrero et al. 2019), we calculated Euclidean distance matrices for each recording frame. We defined 2 individuals as being associated if they were located within a 3.52 cm radius, ie <2 body lengths apart. This distance threshold was determined based on our data, where individuals measured an average of 1.76 cm, making 2 body lengths equal to 3.52 cm. While previous studies have used thresholds of up to 4 body lengths to define shoal membership in fish, including guppies (eg, Pitcher 1993; Croft et al. 2005; Sumpter et al. 2018; Szorkovszky et al. 2018), we applied a stricter threshold to focus on close-range interactions likely to reflect active social or sexual engagement rather than passive proximity.

#### Experiment 3: free-swimming social interactions

The following day, the same cylinder arenas (36 cm in diameter) were used to investigate social interactions in a nonsexual context (see Fig. 1c). Here, 2 same-sex individuals from a single family were introduced to interact with 2 individuals from each of 3 unrelated families. The spatial proximity between each pair of individuals was assessed in the same way that was described in Experiment 2 (Experiment 2: free-swimming mate choice section). In total, 12 trials were performed (each consisting of 8 individuals). Video analyses were conducted by a single observer (A.V.) blind to both sex and relatedness.

#### Measuring morphological traits

One day prior to experiment 1, each fish was photographed with a Canon EOS 800D and all measurements were gathered through *ImageJ* version 1.54. (https://imagej.net/ij/) for subsequent morphological analyses of attractive traits, including body sizeEnd (the distance between the tip of the lower jaw and the caudal peduncle) (mm) and total area of orange coloration (mm^2^) in males (Karino and Matsunaga 2002; Herdman et al. 2004; Corral-López et al. 2017). The difference in body size between stimulus individuals was calculated, as well as the difference in total orange coloration between stimulus males. There was no significant difference in body size between related and unrelated stimulus individuals (Wilcoxon rank-sum test: *W* = 4,413.5, *P* = 0.19). Similarly, total orange coloration did not differ between related and unrelated males (Wilcoxon rank-sum test: *W* = 1,170, *P* = 0.38). The average body length difference was 1.96 mm (±SD, 1.53 mm) in females and 1.06 mm (±0.77 mm) in males. The average body area difference was 15.1 mm^2^ (±13 mm^2^) in females and 6.2 mm^2^ (±4.4 mm^2^) in males. Furthermore, males had an average difference in total area of orange coloration of 1.25 mm^2^ (±1.1 mm^2^).

### Statistical analysis

All statistical analyses were performed using *R* version 4.3.2. We constructed linear and generalized linear mixed effects models (LMMs and GLMMs) using the *lme4* (Bates et al. 2015) and *glmmTMB* (Brooks et al. 2017) packages.

#### Experiment 1: dichotomous mate choice

Strength of preference (SOP) scores (eg, see Chen et al. 2018; Sommer-Trembo et al. 2020) were used to estimate individual preference for either stimuli within each trial. SOP scores were calculated following the equation:


$$\begin{array}{c} \mathrm{SOP}=\frac{T_{R}-T_{\mathrm{UR}}}{T_{R}+T_{\mathrm{UR}}} \end{array}$$

where *T_R_* is the time spent in the related association zone and *T*_UR_ is the time spent in the unrelated association zone. This provides a SOP range from − 1 to 1. A SOP closer to − 1 indicates a preference towards unrelated individuals (ie inbreeding avoidance), a SOP closer to 1 indicates a preference towards related (ie kin preference) and a SOP of 0 indicated an unbiased mating preference.

To determine whether guppies, overall, preferred related or unrelated individuals, a 1-sample *t*-test was performed on the SOP scores obtained from the total duration of the trial. SOP scores were then used as the response variable in an LMM, while sex and mating status were included as fixed factors. A random factor accounted for potential variations due to family origin. SOP scores obtained across multiple choice intervals were additionally calculated to investigate consistent patterns of focal preferences (Table A2). Specifically, we quantified informed choice (IC) by considering association times after the focal individual had visited the association zones of both stimuli individuals. One trial was removed from the IC analysis as the focal individual visited the second stimuli individual late into the video, resulting in a final sample size of 88 trials. Two additional LMMs were performed in each sex to investigate whether differences in attractive traits between related and unrelated stimuli individuals could have influenced focal SOP. In males, the model was fitted with the differences in body length and orange coloration together with mating status as fixed factors while in females, the model was performed with the difference in body length together with mating status as fixed factors. The assumptions of normality and homoscedasticity required for LMMs were checked for all models. All possible 2-way and 3-way interactions among fixed effects were initially included in the models and removed stepwise based on lack of significance, resulting in final models containing only main effects.

#### Experiment 2: free-swimming mate choice

To examine whether virgin and experienced male and female guppies displayed a sexual preference for related or unrelated individuals in a free-swimming arena setting, GLMMs were performed. For each sex, 3 behavioral metrics of mate choice were calculated: orienting, approaching and gliding behaviors in females; and pursuing behaviors, sigmoid displays and mating behaviors in males. In each sex, behavioral data were aggregated at the group level by arena, focal mating status, and relatedness category. This approach was adopted as analyzing behaviors at the dyadic level produced a large number of zero observations (some pairs did not interact during the trial) compromising model fit and inference, even when over-dispersion and zero-inflation were accounted for. Each behavioral metric was analyzed in a separate GLMM, with the total count of that behavior used as the response variable. Focal mating status and relatedness were included as fixed effects, while arena identity was incorporated as a random effect to account for variability between arenas. Mating behaviors included both successful mating acts and forced mating attempts. Individual-level morphological traits (ie, body size and coloration) were not included as predictors, as data were aggregated at the group level. Additionally, we constructed a single GLMM for each sex to evaluate the overall effect of relatedness and mating status on all sexual behaviors (Table 2, d and h). In these models, behavioral type and arena identity were included as random effects. This approach enabled us to estimate the influence of relatedness and mating status on the overall frequency of behaviors while accounting for variations in baseline counts across different behavioral types and arenas. We also examined whether the spatial proximity between all pairs or between female-male pairs only was influenced by their relatedness and respective mating status using a GLMM. As predictors had more than 2 categories, we used the *Anova* function from the *car* package to determine significance effects. Significant main effects were then examined using pairwise Tukey post hoc comparisons in the *emmeans* package. All possible 2-way and 3-way interactions were initially included in all models, but were sequentially excluded from the final models as they were nonsignificant. A negative binomial distribution was used to account for over-dispersion in the data. To account for testing multiple sexual behaviors, we performed a false discovery rate (FDR) correction (Benjamini and Hochberg 1995) using the initial *P*-values reported in the models. We used the function p.adjust and specifying a “fdr” method. We provide the results both before and after FDR correction.

#### Experiment 3: free-swimming social interactions

To examine whether male and female guppies displayed a social preference for related or unrelated individuals in a nonsexual setting, a GLMM was performed with association duration as a response variable. Relatedness and sex were included as fixed effects and arena identity as a random effect to account for variability across arenas. A 2-way interaction term was initially included, but was removed as nonsignificant. A negative binomial distribution was used to account for over-dispersion in the data. To account for testing multiple association durations, we performed a FDR correction (Benjamini and Hochberg 1995) using the initial *P*-values reported in the models.

#### Preference for relatives across experiments

To evaluate whether an individual's preference for relatives remains consistent across experimental designs in a sexual context, we performed Spearman rank correlations between the SOP for relatives in opposite-sex arenas (Experiment 2) and in dichotomous mate choice trials (Experiment 1). Additionally, we assessed the consistency of these preferences across both sexual and nonsexual contexts by performing Spearman rank correlations between (i) SOP for relatives in same-sex arenas (Experiment 3) and dichotomous mate choice trials (Experiment 1) and (ii) SOP for relatives in opposite-sex arenas (Experiment 2) and same-sex arenas (Experiment 3). In Experiment 2, SOP was calculated following Equation 1 with *T_R_* being the time spent in association with the 2 related individuals of the opposite-sex and *T*_UR_ being the time spent in association with the 2 unrelated individuals of the opposite-sex. In Experiment 3, *T_R_* was the time spent in association with the related individual and *T*_UR_ was the time spent in association the 6 unrelated individuals (see Fig. 1c).

**Figure 2:**
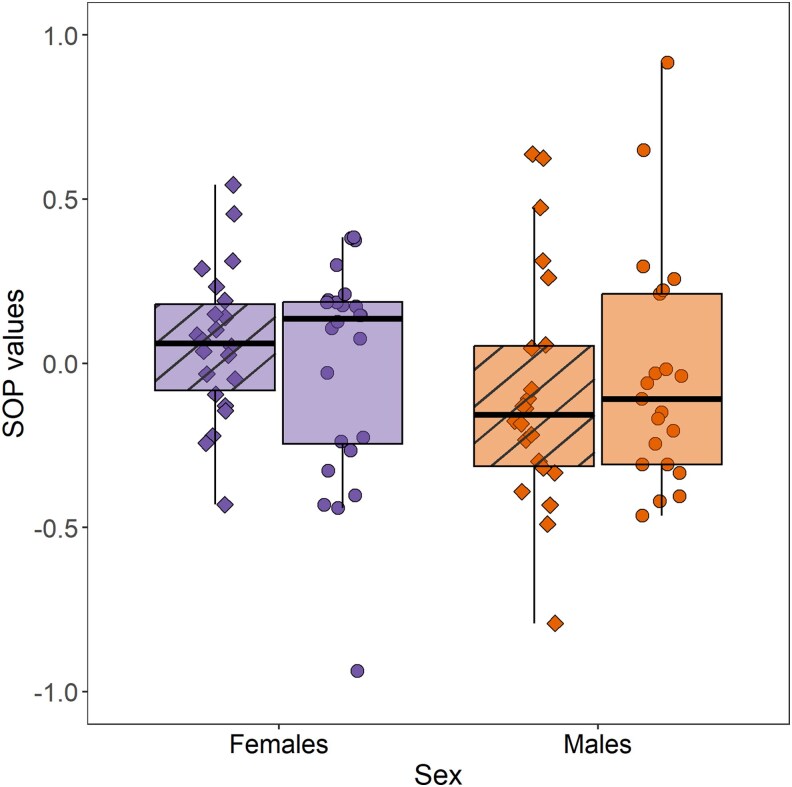
SOP scores obtained in females (purple) and males (orange) throughout the duration of a trial (0 to 30 min). Data are compared between experienced (striped) and virgin (plain) individuals in each sex. In the box plots, the horizontal lines indicate the median (50th percentile) and quartiles (25th and 75th percentiles) and the vertical whiskers indicate the range of the jittered data points.

### Ethical statement

Animal housing and experimental protocols were performed in accordance with the guidelines of Stockholm animal research ethical board (permit number: 18352-2020).

## Results

### Experiment 1: dichotomous mate choice

During the 30-min choice period, females spent an average of 68% of their time in either association zone (mean ± SD: 20.34 ± 3.60 min), while male guppies spent 58% (17.53 ± 4.02 min). On average, guppies did not show a significant overall preference for related or unrelated individuals (mean SOP = −0.016, *t*(*88*) = −0.47, *P* = 0.64). Neither sex nor mating status significantly influenced the SOP for related partners (Table 1, a, Fig. 2). However, related individuals were preferred when they were larger than unrelated individuals. Similar results were obtained when SOP scores were calculated at different choice intervals (Tables A1 and A2). When analyzed separately by sex, SOP for related individuals was also affected by the body size difference between stimuli (Table 1, b and c). In females, the SOP for related males was not influenced by differences in orange coloration between males (Table 1, b). SOP scores obtained across multiple choice intervals were additionally calculated to investigate consistent patterns of focal preferences.

**Table 1:** Effect of sex and mating status on mate preference for related individuals.

Response variable	*N*	Predictors	Estimates [95% CI]	SE	*z*	*P*-values
(a) SOP for relatives	89	Body length	0.05 [0.02;0.09]	0.02	3.23	**0**.002(**)
		Sex (Male)	−0.06 [−0.19;0.05]	0.06	−1.13	0.26
		Mating status (Virgin)	−0.01 [−0.13;0.11]	0.06	−0.18	0.86
(b) Female SOP for related males	*46*	Body length	0.07 [0.01;0.13]	0.03	1.96	0.05(*)
		Orange coloration	0.02 [−0.03;0.07]	0.02	0.94	0.35
		Mating status (Virgin)	−0.05 [−0.18;0.07]	0.06	−0.81	0.43
(c) Male SOP for related females	43	Body length	0.05 [0.01;0.09]	0.02	2.12	0.04(*)
		Mating status (Virgin)	0.06 [−0.09;0.22]	0.08	0.78	0.45

Outputs of linear mixed models (LMMs) investigating (a) the effect of difference in body length, sex and mating status on SOP for related individuals, (b) the effect of difference in body length, difference in orange coloration and mating status on female SOP for related males, and (c) the effect of difference in body length and mating status on male SOP for related females. For each sex, body traits were compared by subtracting the trait value of the unrelated stimuli from that of the related stimuli. Positive differences indicate larger values in the related stimuli, while negative differences indicate larger values in the unrelated stimuli. Estimates represent the fixed-effect regression coefficients (slopes). The total number of trials performed (*N*) is presented for each model, along with the 95% confidence intervals, standard error (SE), test statistic (*z*) and *P-value* for each effect. Significant *P-values* are shown in bold.

### Experiment 2: free-swimming mate choice

#### Female behaviors

**Figure 3:**
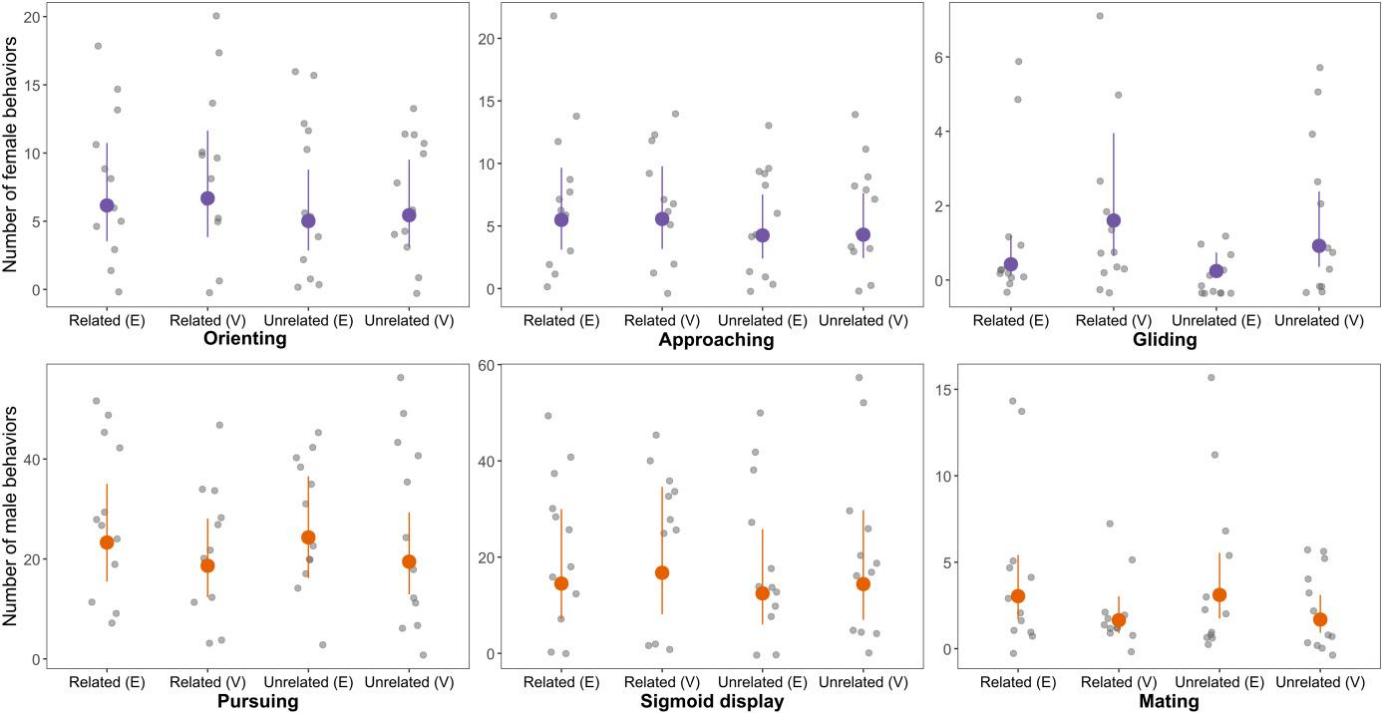
Total number of sexual behaviors observed in females (orienting, approaching and gliding behaviors) and males (pursing behaviors, sigmoid displays and mating behaviors), directed toward related and unrelated individuals in each arena for 30 min. Data are compared between experienced (E) or virgin (V) individuals in each sex (females = purpleand males = orange). Gray points represent raw observations, and colored dots show model-predicted means ± 95% confidence intervals.

**Figure A1:**
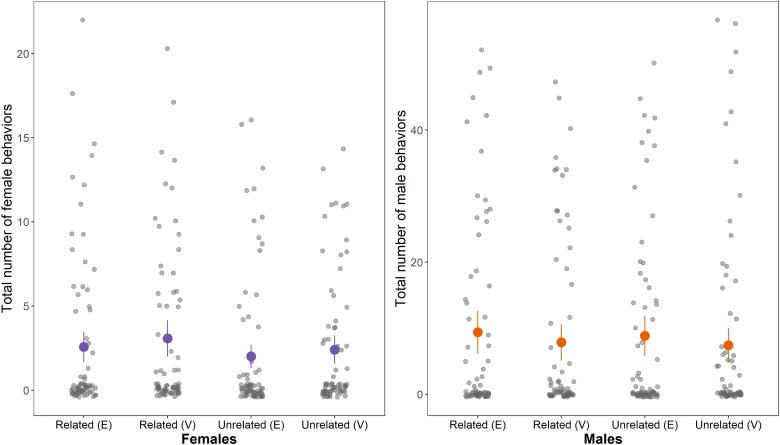
Total number of each sexual behavior observed in females and males, directed toward related and unrelated individuals in each arena for 30 min. Data are compared between experienced (E) or virgin (V) individuals in each sex (females = purple and males = orange). Gray points represent raw observations, and colored dots show model-predicted means ± 95% confidence intervals.

Each of the female behaviors investigated (orienting, approaching, and gliding) did not differ significantly depending on the relatedness to the focal male (GLMMs; Table 2, a, b, and c, Fig. 3). When all sexual behaviors were analyzed together, females directed more responses toward related males in the uncorrected analysis, but this difference did not remain statistically significant after controlling for multiple comparisons (GLMM; Table 2, d, Fig. A1). Additionally, virgin females directed significantly more gliding behaviors toward males than experienced females (GLMM; Table 2, b).

**Table 2:** Effect of relatedness and mating status on female and male sexual behaviors and association duration in mixed-sex arenas.

Response variable	*N*	Predictors	Estimates [95% CI]	SE	*z*	*P*-value	Adj. *P*-value
**Female behaviors**							
(a) Orienting behavior	48	Relatedness (Unrelated)	−0.21 [−0.49;0.08]	0.15	−1.42	0.16	0.21
		Mating status (Virgin)	0.08 [−0.20;0.37]	0.15	0.56	0.57	0.76
(b) Approaching behavior	*48*	Relatedness (Unrelated)	−0.26 [−0.58;0.07]	0.17	−1.54	0.12	0.21
		Mating status (Virgin)	0.01 [−0.32;0.34]	0.17	0.08	0.94	0.94
(c) Gliding behavior	*48*	Relatedness (Unrelated)	−0.55 [−1.46;0.35]	0.46	−1.20	0.23	0.23
		Mating status (Virgin)	1.33 [0.36;2.30]	0.50	2.68	0.007(**)	0.03(*)
(d) **All behaviors**	*48*	Relatedness (Unrelated)	−0.25 [−0.47;−0.02]	0.11	−0.18	0.03(*)	0.12
		Mating status (Virgin)	0.17 [−0.05;0.40]	0.11	1.52	0.13	0.26
**Male behaviors**							
(e) Pursuing behavior	*48*	Relatedness (Unrelated)	0.04 [−0.13;0.22]	0.09	0.46	0.63	0.84
		Mating status (Virgin)	−0.22 [−0.40;−0.04]	0.09	−2.45	0.01(**)	0.02(*)
(f) Sigmoid display	*48*	Relatedness (Unrelated)	−0.15 [−0.49;0.19]	0.18	−0.86	0.39	0.83
		Mating status (Virgin)	0.14 [−0.22;0.51]	0.18	0.78	0.44	0.44
(g) Mating behavior	*48*	Relatedness (Unrelated)	0.02 [−0.42;0.47]	0.23	0.10	0.92	0.92
		Mating status (Virgin)	−0.61 [−1.07;−0.16]	0.23	−2.63	0.008(**)	0.02(*)
(h) **All behaviors**	*48*	Relatedness (Unrelated)	−0.06 [−0.28;0.17]	0.11	−0.50	0.61	0.84
		Mating status (Virgin)	−0.17 [−0.40;0.05]	0.11	−1.53	0.12	0.16
**Association duration**					χ^2^		
(i) Female-male pairs	*192*	Relatedness			0.66	0.42	0.61
		Mating status			11.59	0.003(**)	0.003(**)
(j) All pairs	336	Relatedness			0.25	0.61	0.61
		Mating status			18.41	*<* 0.001(***)	*<* 0.002(***)
		Sex			20.42	*<* 0.001(***)	*<* 0.001(***)

After testing for an effect of relatedness and mating status on each female (a, b, and c) and male (e, f, and g) sexual behaviors in 12 mixed-sex arenas using univariate generalized linear mixed models (GLMMs), we assessed the overall effect of relatedness and mating status by pooling all sexual behaviors into a single GLMM with behavioral type included as a random effect (d and h). For models a to h, we report the *z*-statistic, obtained from the *summary()* function in R. The *z*-statistic is computed as the estimated coefficient divided by its standard error (SE) and assesses the significance of each fixed-effect in the model. For GLMMs on association durations (i and j), we used the *Anova* function from the *car* package to obtain significance effects as predictors have more than 2 categories and reported the χ^2^ test statistic from likelihood ratio tests. Significant effects were further examined using pairwise Tukey post hoc comparisons in the *emmeans* package, as described in the main text. Estimates represent the fixed-effect regression coefficients (slopes). The total number of individuals or pairs assessed (*N*) is presented for each model, along with the 95% confidence intervals, SE, test statistic (*z* or χ^2^ value), uncorrected *P-value*, and FDR-corrected *P-value* (*Adj. P-value*) for each effect. Significant *P-values* are shown in bold.

#### Male behaviors

Similarly to females, none of the male behaviors investigated separately (pursuing behaviors, sigmoiod displays and mating behaviors), significantly differed depending on relatedness to the focal male (GLMMs; Table 2, e, f and g, Fig. 3). However, virgin males performed significantly fewer pursuing and mating behaviors than experienced males (GLMMs; Table 2, f and g, Fig. 3). When all sexual behaviors were analyzed, males did not significantly bias their behaviors depending on relatedness or mating status (GLMM; Table 2, h, Fig. A1).

#### Association duration

The frequency of female-male pair associations did not significantly differ based on the relatedness of the individuals involved (Table 2, i). However, mating status had a significant effect on the frequency of female-male pair associations (Table 2, i). Specifically, virgin individuals associated less frequently compared with pairs of experienced individuals (*z* = −2.98, *P* = 0.008) or virgin-experienced individuals (*z* = −3.06, *P* = 0.006).

The same pattern was observed when investigating the frequency of all pair associations (Table 2, j). The relatedness did not influence the frequency of associations (Table 2, j), but virgin individuals appeared to associate less frequently compared with pairs of experienced individuals (*z* = −3.76, *P* < 0.001) or virgin-experienced individuals (*z* = −3.89, *P* < 0.001). The sex of the individuals involved also significantly influenced the association frequency (Table 2, j). Male-male pairs associated significantly less than both male-female pairs (*z* = −4.25, *P* < 0.001) or female-female pairs (*z* = −3.73, *P* < 0.001).

### Experiment 3: free-swimming social interactions

**Figure 4:**
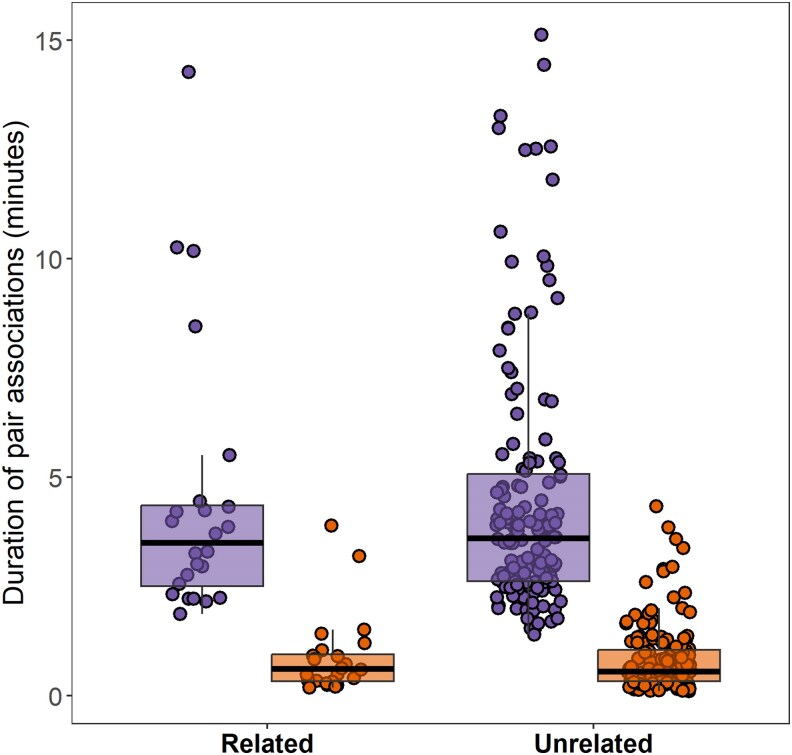
Association duration (min) between related and nonrelated pairs in same-sex arenas (females = purple, males = orange) analysed for 30 min. In the box plots, the horizontal lines indicate the median (50th percentile) and quartiles (25th and 75th percentiles) and the vertical whiskers indicate the range of the jittered data points.

The frequency of pair associations did not significantly differ based on the relatedness of the individuals involved (Table 3, Fig. 4). However, males associated significantly less frequently compared with female pairs (Table 3, Fig. 4).

**Table 3:** Effect of relatedness and sex on association duration in same-sex arenas.

Response variable	*N*	Predictors	Estimates [95% CI]	SE	*z*	*P*-values	Adj. *P*-values
Association duration	*336*	Relatedness (Unrelated)	−0.05 [−0.21;0.10]	0.08	−0.66	0.51	0.61
		Sex (Male)	−1.65 [−2.18;−1.12]	0.27	−6.09	*<* 0.001(***)	*<* 0.001(***)

We evaluated the effect of relatedness and sex on association duration in 12 same-sex arenas using a GLMM with arena type included as a random effect. Estimates represent the fixed-effect regression coefficients (slopes). The total number of pairs assessed (*N*) is presented along with the 95% confidence intervals, standard error (SE), test statistic (*z*), uncorrected *P-value*, and FDR-corrected *P-value* (*Adj. P-value*) for each effect. Significant *P-values* are shown in bold.

### Preference for relatives across experiments

**Figure 5:**
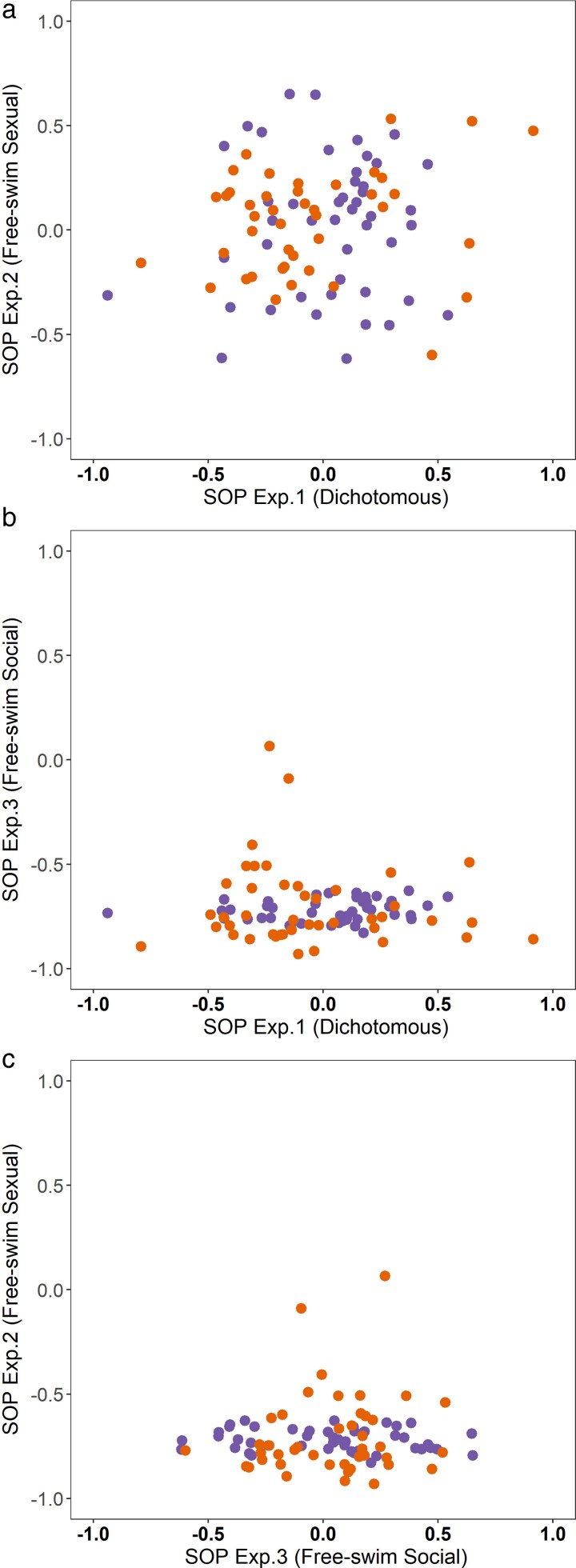
Pairwise relationship between a) SOP for opposite-sex relatives in free-swimming arenas (Experiment 2) and SOP for opposite-sex relatives in dichotomous mate choice trials (Experiment 1), b) SOP for relatives in same-sex arenas (Experiment 3) and SOP for opposite-sex relatives in dichotomous mate choice trials (Experiment 1), c) SOP for opposite-sex relatives in free-swimming arenas (Experiment 2) and SOP for relatives in same-sex arenas (Experiment 3). Data points represent individual metrics (females = purple, males = orange).

In both males and females, no significant correlation was observed between the SOP for opposite-sex relatives in free-swimming arenas and in dichotomous mate choice trials (females: *r*_46_ = 0.005, *P* = 0.97; males: *r*_43_ = 0.17, *P* = 0.28; Fig. 5a), indicating an individual's preference for relatives is not consistent across experimental designs in a sexual context. Similarly, SOP for relatives in same-sex arenas did not correlate significantly with SOP in dichotomous mate choice trials (females: *r*_46_ = 0.24, *P* = 0.11; males: *r*_43_ = −0.08, *P* = 0.60; Fig. 5b) or in opposite-sex arenas (females: *r*_46_ = −0.14, *P* = 0.36; males: *r*_43_ = 0.11, *P* = 0.49; Fig. 5c), suggesting that an individual's preference for relatives is not consistent across sexual and nonsexual contexts.

## Discussion

Contrary to longstanding expectations that animals should avoid mating with kin to reduce the risk of inbreeding depression, we found no evidence of precopulatory inbreeding avoidance in guppies. Across both dichotomous mate choice and free-swimming experiments, females and males did not prefer unrelated over related partners. Despite differences in sexual behaviors between virgin and mated individuals, we found no evidence that mating status influenced mate preference for related versus unrelated mates. Both sexes showed consistent preferences for larger individuals in dichotomous mate choice trials, suggesting body size may play a more prominent role in mate choice than genetic relatedness. Moreover, the overall absence of inbreeding avoidance observed in our study is not explained by nonsexual kin affiliation, as same-sex pair associations were not influenced by relatedness.

Regardless of their mating status, female guppies did not display a clear preference for unrelated over related partners. In the free-swimming experiment, females appeared slightly more responsive to male courtship from related males, although this trend was not statistically significant after correcting for multiple tests. While inbreeding depression is expected to drive selection for inbreeding avoidance behaviors (Blouin and Blouin 1988; Pusey and Wolf 1996), these costs may be offset by indirect inclusive fitness benefits gained through mating with kin. Kin preference through mate choice has been mainly documented in species that do not experience inbreeding depression such as the African cichlid fish (*Pelvicachromis taeniatus*) (Thünken et al. 2007), the ground tit (*Parus humilis*) (Wang and Lu 2011) and the white's skink (*Liopholis whitii*) (Bordogna et al. 2016). Interestingly, biased mating preference towards kin has also been found in species suffering from inbreeding depression including the American crow (Townsend et al. 2019) and the fruit fly (*Drosophila melanogaster*) (Ehiobu et al. 1989; Loyau et al. 2012). Nevertheless, we observed no clear differences in association duration or sexual behaviors between related and unrelated pairs. The trend of increased responsiveness to related males in the free-swimming context suggests that social environment might influence mate assessment. In more complex social settings, females might have reduced stress associated with social isolation or be influenced by the presence and behavior of other individuals (eg, mate choice copying, Dugatkin and Godin 1992; Godin et al. 2005). While speculative, these factors could help explain why any weak kin-directed preference may appear only under free, socially complex conditions. We note that Experiments 2 and 3 were based on a relatively small number sample size (*n* = 12), which may reduce statistical power and prevent detection of subtle effects. However, this level of replication is consistent with previous high-resolution behavioral studies of fish social dynamics (eg, Herbert-Read et al. 2011; Schaerf et al. 2024), which similarly analyzed fine-scale interactions within complex social groups. Future studies with larger samples could clarify whether the slight trend of female responsiveness to related males reflects an adaptive inclusive fitness strategy (Parker 1979) or instead arises from other social dynamics.

In virgin females, our results align with previous mate choice experiments that reported no female preference for unrelated males through phenotype matching, using either dichotomous choice trials (Viken et al. 2006; Guevara-Fiore et al. 2010; Zajitschek and Brooks 2010) or free-swimming arenas (Pitcher et al. 2008; Daniel and Rodd 2016). Although virgin females did not exhibit reduced choosiness toward related males, they performed more gliding behaviors overall, a behavior that indicates acceptance of a displaying male and is commonly observed immediately prior to mating (Liley 1966; Houde 1997c), suggesting higher baseline responsiveness to male courtship regardless of relatedness. It is worth noting that virgin females in free-swimming arenas had already been exposed to males during the dichotomous choice trials. Although they remained unmated, prior exposure to male courtship could potentially influence responsiveness. However, virgin females still performed more gliding behaviors overall than experienced females, suggesting that the higher baseline responsiveness observed is robust to any effects of previous male encounters. This finding is partly consistent with theoretical models predicting that virgins may be less choosy and more responsive to male courtship, as rejecting a mating opportunity carries the risk of dying unmated (Jennions and Petrie 2000; Kokko and Ots 2006). However, we found no evidence that experienced females shifted their mate preferences toward unrelated males, in either the free-swimming or dichotomous mate choice experiments. Thus, our results suggest that female guppies do not adjust inbreeding avoidance based on mating status. This contrasts with the findings of Daniel and Rodd (2016), who reported that experienced females preferred unrelated males, while virgins did not display any preference. While theory would predict that experienced females might choose mates based on indicators of higher quality, such as genetic relatedness, our results instead align with meta-analyses reporting no consistent evidence that virgin females are less choosy than mated females (Richardson and Zuk 2023) or that mating status influences inbreeding avoidance (de Boer et al. 2021). Finally, whereas Daniel and Rodd (2016) study relied on live behavioral observations, we used video recordings and automated tracking to account for individual identities, potentially providing more precise quantification of female mate preferences.

Male guppies, irrespective of their mating status, exhibited no consistent preference based on relatedness in either experimental setup. The overall absence of strong sexual preferences in males aligns with theoretical expectations that, as the mate-limited sex, males prioritize reproductive opportunities over mate selectivity (Dosen and Montgomerie 2004). Theoretical models (Parker 1979; Waser et al. 1986; Kokko and Ots 2006; Parker 2006; Puurtinen 2011) emphasize that inbreeding tolerance might be adaptive in males even when inbreeding depression occurs due to the opportunity cost in terms of missed opportunities to outbreed. This is particularly true for poeciliid fishes, where reproductive success is strongly tied to the number of mates acquired (Blouin and Blouin 1988). Moreover, the evolution of male inbreeding avoidance behaviors in guppies may mainly be driven by female mate choice, as it was shown in polygynous primates (Tennenhouse 2014). Here, we did not detect female mate preference towards unrelated males which additionally limits the selective pressures that might favor male inbreeding avoidance in guppies.

The benefits of nonsexual interactions among kin may limit the evolution of precopulatory inbreeding avoidance (Dorsey and Rosenthal 2023). In salmonids (*Salmo salar* and *Oncorhynchus mykiss*), juveniles in shoals of related individuals grow faster and display fewer antagonistic behaviors than do shoals of unrelated individuals (Brown and Brown 1996). Similarly, growth was higher in kin-only shoals of juvenile *Pelvicachromis taeniatus* than in mixed groups, indicating fitness benefits of kin shoaling. In guppies, Hain and Neff (2007) found that juveniles preferred to shoal with half-siblings than with unrelated individuals in the laboratory, while significant kin structure was found by Piyapong et al. (2011) in wild juvenile guppy shoals, although only in high predation environments. Guppies may therefore gain potential benefit from shoaling preferentially with kin at the juvenile stage. Here, we found no kin structure in pair associations within same-sex arenas, suggesting that nonsexual cooperation among adult kin is not a mechanism underlying the general absence of precopulatory inbreeding avoidance seen in this study. This may be explained by the reduced occurrence of shoaling behaviors performed by adults in natural populations. Although shoaling is a common defense against fish predation at the adult phase (Pitcher 1993; Croft et al. 2006), previous studies consistently showed no evidence of kin structure in adult guppy aggregations of populations subjected to high predation pressure (Russell et al. 2004; Hain and Neff 2007) where shoaling propensities should be maximized.

Precopulatory inbreeding avoidance mechanisms may also not evolve in guppies if they depend on sperm competition and cryptic female choice to mitigate the risks of inbreeding. The potential for such postcopulatory avoidance mechanisms is high in guppies as they display a highly polygynandrous mating system (Evans and Magurran 1999), with females mating with several males each time they are receptive and males engaging in forced copulations (Magurran and Seghers 1994; Pilastro and Bisazza 1999). Moreover, females can store sperm for several months (Liley 1966; Houde 1997c), and guppies exhibit last male sperm precedence (Evans and Magurran 2001). There is some evidence that postcopulatory avoidance mechanisms could act as a filter against incompatible sperm and minimize fertilization by sperm from mating partners with overly similar genotypes (Gasparini and Pilastro 2011; Fitzpatrick and Evans 2014), although counter examples show no bias fertilization toward less closely related males during competitive fertilizations (Evans et al. 2008; Pitcher et al. 2008). The overall prevalence of both pre and postcopulatory inbreeding avoidance mechanisms may also depend on the severity of inbreeding depression that may be different between wild populations or laboratory raised populations. In addition, males often migrate in their natural environment (Croft et al. 2003), lowering the chance of meeting a relative to mate with, which may also reduce the propensity of inbreeding avoidance in guppies. Although our fish are laboratory raised, the expression of inbreeding avoidance before mating might still be low due to conserved selective pressures.

In conclusion, our findings highlight a limited role for precopulatory mechanisms in inbreeding avoidance among guppies. Potential female preferences for related mates in certain contexts may reflect kin selection benefits, while males appear to prioritize reproductive opportunities over relatedness. Furthermore, the absence of kin-structured associations in same-sex arenas indicates that examining nonsexual interactions offer little insight into resolving the inbreeding paradox. Together, these results indicate that dispersal and postcopulatory processes may be more important than premating selection in mitigating the costs of inbreeding depression in guppies.

## Data Availability

The data and scripts underlying this article are available on Zenodo, at https://doi.org/10.5281/zenodo.17830066.

## Appendix

**Table A1:** The effect of difference in body length, sex and mating status on the strength of preference (SOP) for related individuals when considering multiple time intervals.

Response variable	*N*	Predictors	χ^2^	*P-values*
*SOP for relatives*	89	Body length	14.16	**<0**.**001(***)**
		Sex	2.01	0.16
		Mating status	0.02	0.89
		Time interval	3.30	0.19

Outputs of a linear mixed models (LMM) investigating the effect of difference in body length, sex, and mating status on SOP for related individuals when considering multiple time intervals. For each sex, body traits were compared by subtracting the trait value of the unrelated stimuli from that of the related stimuli. SOP scores comparisons were done across multiple time intervals: first 10 min, 10 to 20 min, and 20 to 30 min. The total number of trials performed (*N*) is presented, along with the test statistic (*χ*^2^) and *P-value* for each effect. Significant *P-values* are shown in bold

**Table A2:** The effect of sex and mating status on the strengh of preference (SOP) for related individuals across multiple time intervals.

Intervals	*N*	Predictors	Estimates	SE	*z*	*P-values*
*0 to 10 min*	89	Sex (Male)	−0.10	0.08	−1.12	0.26
		Mating status (Virgin)	0.001	0.09	0.02	0.99
*10 to 20 min*	*89*	Sex (Male)	−0.11	0.09	−1.29	0.20
		Mating status (Virgin)	−0.01	0.09	−0.16	0.87
*0 to 20 min*	*89*	Sex (Male)	−0.11	0.07	−1.64	0.11
		Mating status (Virgin)	0.001	0.07	0.01	0.99
*20 to 30 min*	*89*	Sex (Male)	−0.07	0.11	−0.63	0.53
		Mating status (Virgin)	−0.01	0.11	−0.01	0.99
*0 to end (IC)*	*88*	Sex (Male)	−0.08	0.06	−1.32	0.19
		Mating status (Virgin)	−0.002	0.06	−0.03	0.97
*0 to 10 min(IC)*	*88*	Sex (Male)	−0.17	0.08	−2.18	**0**.**03(*)**
		Mating status (Virgin)	0.01	0.08	0.13	0.90
*10 to 20 min(IC)*	*88*	Sex (Male)	−0.05	0.09	−0.57	0.57
		Mating status (Virgin)	−0.03	0.09	−0.36	0.72

Outputs of a linear mixed models (LMM) investigating the effect of difference in body length, sex, and mating status on SOP for related individuals. For each sex, body traits were compared by subtracting the trait value of the unrelated stimuli from that of the related stimuli. SOP scores comparisons were done across multiple time intervals: first 10 min, 10 to 20 min, first 20 min, 20 to 30 min, overall time after IC, first 10 min after IC, 10 to 20 after IC. Estimates represent the fixed-effect regression coefficients (slopes). The total number of trials performed (*N*) is presented for each model, along with the 95% confidence intervals, standard error (SE), test statistic (*z*) and *P-value* for each effect. Significant *P-values* are shown in bold.
